# Persistent capillary rarefication in long COVID syndrome

**DOI:** 10.1007/s10456-022-09850-9

**Published:** 2022-08-11

**Authors:** Irina Osiaevi, Arik Schulze, Georg Evers, Kimon Harmening, Hans Vink, Philipp Kümpers, Michael Mohr, Alexandros Rovas

**Affiliations:** 1 Department of Medicine A, Hematology, Oncology and Pulmonary Medicine, University Hospital Münster, 48149 Münster, Germany; 2 Department of Physiology, Cardiovascular Research Institute Maastricht, Maastricht University, Maastricht, The Netherlands; 3 Department of Medicine D, Division of General Internal and Emergency Medicine, Nephrology, and Rheumatology, University Hospital Münster, Albert-Schweitzer-Campus 1, 48149 Münster, Germany

**Keywords:** COVID-19, Long COVID microcirculation, Endotheliopathy, Sublingual microscopy, Endothelial glycocalyx, Microvascular health score

## Abstract

**Background:**

Recent studies have highlighted Coronavirus disease 2019 (COVID-19) as a multisystemic vascular disease. Up to 60% of the patients suffer from long-term sequelae and persistent symptoms even 6 months after the initial infection.

**Methods:**

This prospective, observational study included 58 participants, 27 of whom were long COVID patients with persistent symptoms > 12 weeks after recovery from PCR-confirmed SARS-CoV-2 infection. Fifteen healthy volunteers and a historical cohort of critically ill COVID-19 patients (*n* = 16) served as controls. All participants underwent sublingual videomicroscopy using sidestream dark field imaging. A newly developed version of Glycocheck™ software was used to quantify vascular density, perfused boundary region (PBR-an inverse variable of endothelial glycocalyx dimensions), red blood cell velocity (VRBC) and the microvascular health score (MVHS™) in sublingual microvessels with diameters 4–25 µm.

**Measurements and main results:**

Although dimensions of the glycocalyx were comparable to those of healthy controls, a µm-precise analysis showed a significant decrease of vascular density, that exclusively affected very small capillaries (D5: − 45.16%; D6: − 35.60%; D7: − 22.79%). Plotting VRBC of capillaries and feed vessels showed that the number of capillaries perfused in long COVID patients was comparable to that of critically ill COVID-19 patients and did not respond adequately to local variations of tissue metabolic demand. MVHS was markedly reduced in the long COVID cohort (healthy 3.87 vs. long COVID 2.72 points; *p* = 0.002).

**Conclusions:**

Our current data strongly suggest that COVID-19 leaves a persistent capillary rarefication even 18 months after infection. Whether, to what extent, and when the observed damage might be reversible remains unclear.

**Supplementary Information:**

The online version contains supplementary material available at 10.1007/s10456-022-09850-9.

## Introduction

Coronavirus disease 2019 (COVID-19) is a systemic infectious disease caused by severe acute respiratory syndrome coronavirus 2 (SARS-CoV-2) [1]. Recent studies have highlighted COVID-19 as a multisystemic vascular disease associating with severe microvascular impairment and endothelial injury [2–4]. Up to 60% of the patients suffer from long-term sequelae and persistent symptoms of various intensity even 6 months after the initial infection, which can severely impair their performance and quality of life [5]. These symptoms include exertional dyspnea as well as dyspnea at rest, palpitations, fatigue, and mental and cognitive disorders. The pathophysiological hallmark of post-COVID symptoms remains still poorly understood [6]. However, possible causes might include disturbed systemic microvasculature. Consequently, we performed this prospective pilot study to characterize microvascular changes in patients suffering long COVID symptoms.

## Materials and methods

### Study population and study design

This prospective, observational study was conducted from January to December 2021 in the Division of Pulmonary Medicine at University Hospital Münster. We enrolled 27 adults with long-haul COVID (alpha variant) referred to our outpatient clinic due to persistent symptoms > 12 weeks after recovery from PCR-confirmed SARS-CoV-2 infection. Alternative diagnoses were ruled out in all patients by thorough examination [7]. Fifteen apparently healthy, age- and sex-matched volunteers, as well as a historical cohort of hospitalized COVID-19 individuals (*n* = 16, alpha variant) with critical disease [4] served as negative and positive controls, respectively.

All participants underwent sublingual video microscopy as part of their clinical evaluation by an experienced physician. Exclusion criterion was inflammation of the oral mucosa, which could affect the sublingual microvasculature. The study was approved by the competent ethics committee (Ref. 2020-585-f-S & amendments of Ref. 2016-073-f-S) and was performed in accordance with the Declaration of Helsinki. Written informed consent was obtained.

### In vivo assessment of the sublingual microcirculation and glycocalyx dimensions

A sidestream dark field (SDF) camera (CapiScope HVCS, KK Technology, Honiton, UK) coupled to the GlycoCheck™ software (Microvascular Health Solutions Inc., Alpine, UT, USA) was used to visualize the sublingual microvasculature and record the movement of red blood cells (RBC) in the microvessels with a diameter (D) of 4–25 µm, as previously described in detail [8]. Briefly, the following parameters were calculated:

*Perfused boundary region* (PBR, in µm), an estimate of the dynamic lateral movement of RBCs into the permeable part of the endothelial glycocalyx layer. The higher the PBR values, the more diminished the glycocalyx dimensions.

*RBC velocity* (VRBC, in µm/s), calculated by dividing longitudinal RBC displacement by the time between video frames.

*Capillary density* (in 10^−2^ mm/mm^2^), defined as the vascular density of vessels with a diameter ≤ the diameter of a single RBC (D ~ 7–8 µm [9]); capillary density D < 7 µm.

*Microvascular health score* (MVHS™, in points), a novel score that combines microcirculatory impairment and eGC dimensions; higher values indicate healthier microvasculature. The MVHS is calculated by dividing capillary blood volume (CBV; i.e., capillary density multiplied by segment-specific capillary cross-sectional area ($\pi$ * radius^2^) by the PBR value.

*Capillary recruitment* (CR, in %), an estimate of the microvascular ability to recruit additional capillaries under stress conditions. CR can be derived *per group* by the slope of the relationship between VRBC in the capillaries (≤ 7 µm) and in the feed vessels (≥ 10 µm) and is defined as 1 − slope [VRBC (D ≤ 7 µm), VRBC (D ≥ 10 µm)].

### Statistical analysis

Data are presented as absolute numbers, percentages, and medians with corresponding 25th and 75th percentiles (interquartile range; IQR), as appropriate. Parameters between groups were compared with the non-parametric Mann–Whitney *U* test, the chi-square test or Kruskal–Wallis test. To adjust for multiple testing in comparisons of microcirculatory parameters per diameter class, the false discovery rate (FDR) approach of Benjamini, Krieger, and Yekutieli was used setting a *q*-value < 0.05 as significant. Spearman rank correlation coefficient (rs) was used to assess correlations between variables. Linear regression was used to assess the association between MVHS and demographic variables. Applied tests were two-sided, and statistical significance was set to *p* < 0.05. SPSS version 26 (IBM Corporation, Armonk, NY, USA) and GraphPad Prism version 8.4.3 (GraphPad Prism Software Inc., San Diego, CA, USA) were used for statistical analyses and preparation of figures.

## Results

The long COVID cohort encompasses 27 adult patients, studied after a median (IQR) duration of 541 (305–569) days post-COVID-19 infection. The patients (55.5% men), aged 53 (39–51) years and had a median Charlson Comorbidity Index (CCI) of 0 (Table 1).

**Table 1:** Baseline characteristics

Variable	Healthy controls	Historical ICU COVID-19 cohort	Long COVID cohort	*p* value^#^	*p* value^++^
Number of participants (*n*; %)	15	16	27	–	–
Female sex (*n*; %)	8 (53.3)	3 (13)	12 (44.4)	0.75	**0.03**
Age [years, median (IQR)]	53 (39–69)	62 (56–72)	53 (39–51)	0.45	**0.004**
BMI [kg/m^2^, median (IQR)]	23.7 (22.0–25.2)	27.3 (23.5–31.5)	27.1 (23.2–29.1)	0.17	0.74
Hospitalized (*n*; %)	–	16 (100)	15 (55.5)	–	–
SOFA score [pts, median (IQR)]	–	6 (2–13)	–	–	–
Vasopressors (*n*; %)	–	6 (37.5)	–	–	–
Invasive mechanical ventilation (*n*; %)	–	14 (87.5)	–	–	–
In-hospital mortality (*n*; %)	–	6 (26.1)	–	–	–
Days after COVID-19 infection	–	7 (1–17)	541 (305–569)	–	** < 0.001**
CCI score [median (IQR)]	0	1 (0–3)	0 (0)	0.99	**0.005**
Comorbidities (*n*; %)					
Arterial hypertension	0	11 (68.8)	4 (14.8)	0.28	** < 0.001**
Diabetes mellitus	0	0	0	–	–
Chronic respiratory disease	0	3 (18.8)	2 (7.4)	0.53	0.34
Congestive heart failure	0	6 (37.5)	0	0.99	**0.001**
Rheumatologic disease	0	3 (17.2)	3 (11.1)	0.54	0.65
Malignancy	0	3 (18.8)	1 (3.7)	0.99	0.14
Long COVID symptoms (*n*; %)					
> 1 Symptoms	–	–	23 (85.2)	–	–
Fatigue/Weakness	–	–	23 (85.2)	–	–
Dyspnea	–	–	22 (81.5)	–	–
Chest discomfort	–	–	6 (22.2)	–	–
Neurocognitive dysfunction	–	–	4 (14.8)	–	–
Persistent cough	–	–	2 (7.4)	–	–
Headache	–	–	2 (7.4)	–	–
Sublingual videomicroscopy [median (IQR)]					
PBR (µm)	2.23 (2.02–2.31)	2.39 (2.13–2.52)	2.15 (2.03–2.27)	0.45	**0.02**
Capillary density (10^−2^ mm/mm^2^)	77.89 (66.66–83.27)	19.88 (12.13–42.08)	46.43 (39.15–63.49)	** < 0.001**	** < 0.001**
VRBC_4–7 µm_ (µm/sec)	103 (89.87–112.6)	82.67 (75.03–95.53)	114.9 (102.7–134.4)	**0.01**	** < 0.001**
VRBC_≥10 µm_ (µm/sec)	100.60 (93.95–110.10)	85.14 (97.12–69.55)	124 (119.60–132.20)	** < 0.001**	** < 0.001**
MVHS (points)	3.87 (3.15–4.87)	0.92 (0.766–1.85)	2.72 (2.21–3.01)	**0.002**	** < 0.001**
Capillary recruitment (%)*	70	17	29	–	–

Bold indicates the statistically significant p values (p < 0.05)

*BMI* Body mass index, *CBV* capillary blood volume, *CCI score* Charlson Comorbidity Index, *COVID*-19 Coronavirus disease 2019, *CRP* C-reactive protein, *D* capillary diameter, *IQR* interquartile range, *MVHS* Microvascular Health Score, *PBR* Perfused boundary region, *PCT* Procalcitonin, *SOFA score* Sequential Organ Failure Assessment score, *V*_*RBC*_ red blood cell velocity

*Capillary recruitment was estimated using 1 − slope (*V*_RBC_ (*D* ≤ 7 µm), *V*_RBC_ (*D* ≥ 10 µm)) in a per group analysis

^#^*p* values were calculated between healthy controls and long COVID patients

^++^*p* values were calculated between historical COVID-19 cohort and long COVID patients

**Fig. 1:**
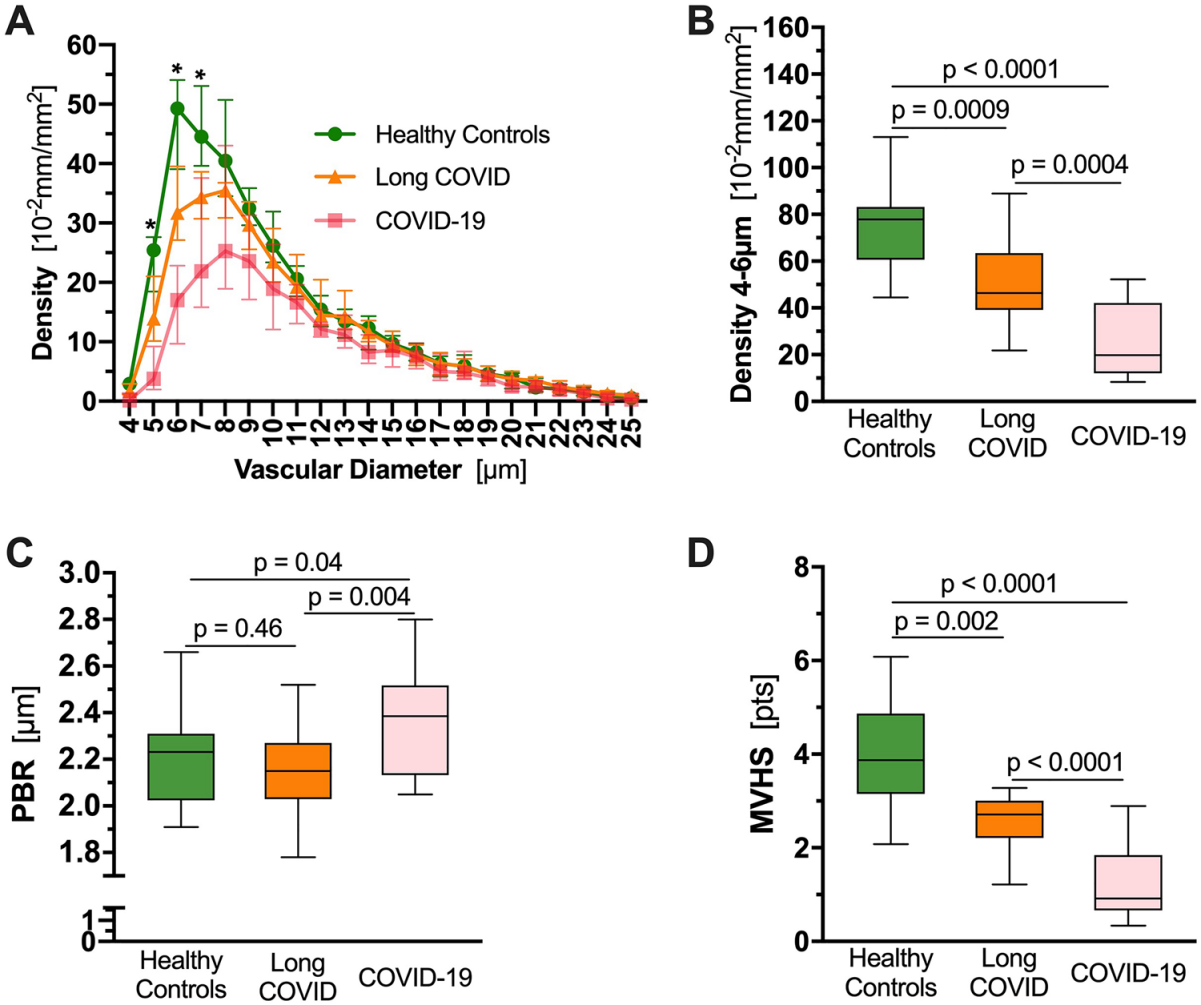
Microvascular phenotyping by quantitative sublingual video microscopy. **A** Median and IQR values of vascular density of healthy controls (green), and long-haul COVID-19 (orange) patients based on the diameter class from 4 to 25 µm. A historical cohort of hospitalized COVID-19 patients (pink) is shown for reference. *q < 0.05, **q < 0.01, ***q < 0.001. **B**–**D** Boxplots of **B** capillary density (*D*_4–6 µm_) and **C** PBR_4–25 µm_ and **D** MVHS™ of healthy controls (green), long-haul COVID individuals (orange), COVID-19 patients (pink). *COVID*-19 Coronavirus disease 2019, *CR* capillary recruitment, *D* diameter, *PBR* perfused boundary region, *RBC* red blood cell, *VRBC* red blood cell velocity, *MVHS*™ microvascular health score

Long COVID patients showed a significantly lower vascular density compared to healthy controls. Specifically, a µm-precise analysis showed the decrease in vascular density to exclusively affect very small capillaries (D5: − 45.16%; D6: − 35.60%; D7: − 22.79%) (Fig. 1A). Compared with healthy controls, the capillary density of long COVID patients was decreased by 41% (Fig. 1B). However, glycocalyx dimensions did not differ from those of the healthy volunteers (Fig. 1C), even in a µm-precise analysis (data not shown). Upon recombination of these indices into MVHS™, severe impairment of the microcirculation in individuals with long COVID [healthy 3.87 (3.15–4.87) vs. long COVID 2.72 (2.21–3.01) points, *p* = 0.002; Fig. 1D] is emphasized. In a pooled analysis of the measurements of all study participants (*n* = 58), disease group (healthy controls, long COVID, historical acute COVID-19 cohort) was the only variable associated with the MVHS™. This association remained significant after adjustment for age, sex, CCI and BMI (β = − 0.52, *p* < 0.001).

**Fig. 2:**
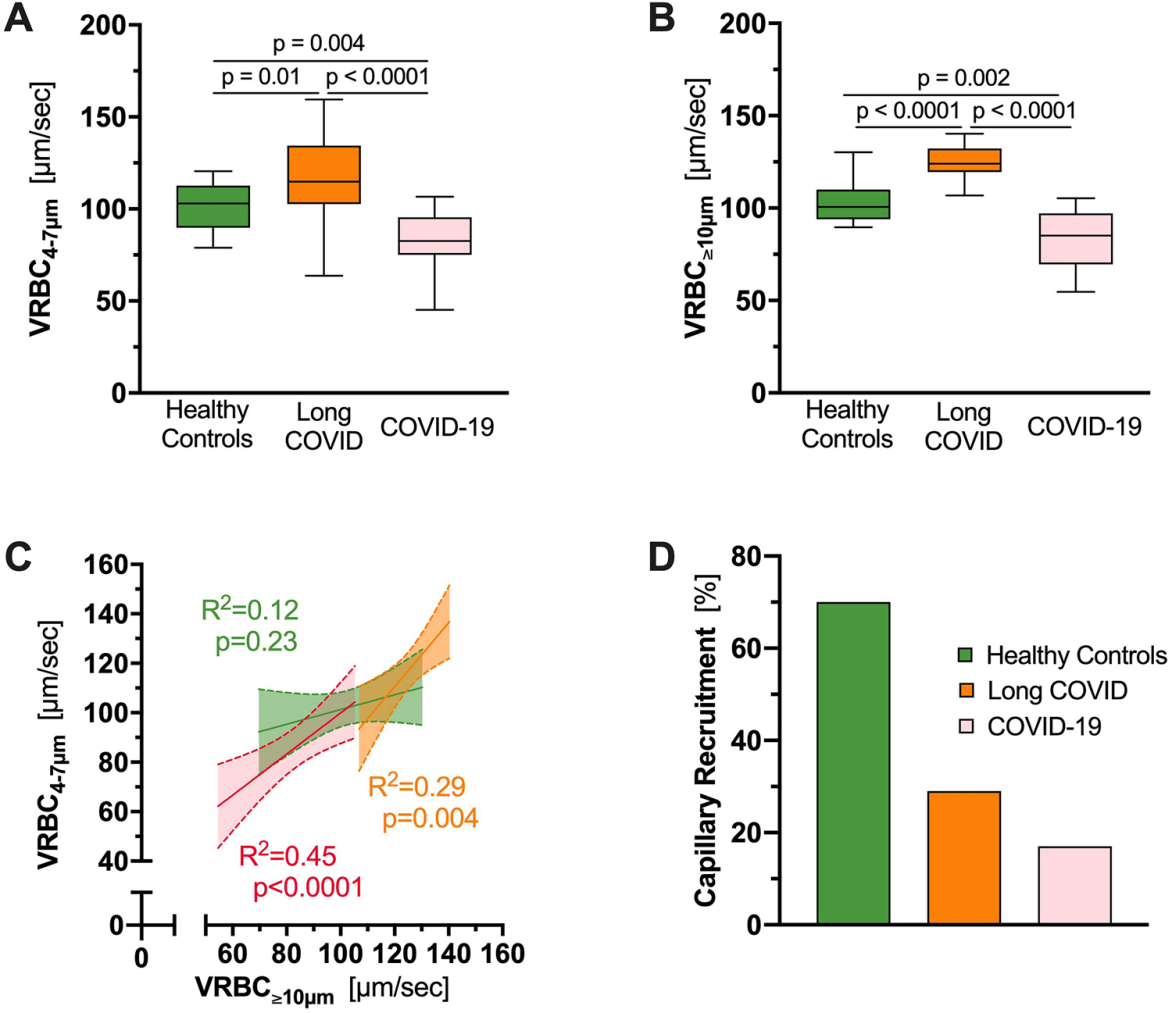
Red blood cell velocities and recruitment capacity. **A**, **B** Boxplots of **A** VRBC in capillaries (*D* ≤ 7 µm) and **B** VRBC in feed vessels (*D* ≥ 10 µm) of long COVID individuals (orange), hospitalized COVID-19 patients (pink) and healthy controls (green). **C** Scatter dot plots and simple linear regression (slope) with 95% confidence intervals of VRBC in capillaries plotted against VRBC in feed vessels of long COVID individuals (orange), hospitalized COVID-19 patients (pink) and healthy controls (green). **D** Bar charts showing the capillary recruitment (CR), defined as 1 − slope [VRBC (*D* ≤ 7 µm) vs. VRBC (*D* ≥ 10 µm)] per group

We previously used a ratio derived from VRBC in feed vessels relative to capillaries (VRBC (D ≥ 10 µm)/VRBC (D ≤ 7 µm) to calculate capillary recruitment (see above) [8]. Analysis of RBC velocity (VRBC) revealed significant differences between all three study groups, both in VRBC_4–7 µm_ (Fig. 2A) and in VRBC_≥10 µm_ (Fig. 2B). Surprisingly, VRBC was significantly higher in capillaries as well as feed vessels in long COVID patients compared with healthy individuals (VRBC_4–7 µm_: 114.9 [102.7–134.4] vs. 103 [89.87–112.6] µm/s, *p* = 0.01; VRBC_≥10 µm_: 124 [119.6–132.2] vs. 100.6 [93.95–110.1] µm/s; *p* < 0.0001). Plotting VRBC_≥10 µm_ vs. VRBC_4–7 µm_ revealed a strong dependency between capillaries and feed vessels in both, individuals with long COVID and hospitalized COVID-19 patients (long COVID: *R*^2^ = 0.29, *p* = 0.004; COVID-19: *R*^2^ = 0.45, *p* < 0.0001), indicating impaired capillary (de-)recruitment in these groups. In contrast, capillary VRBC was constant in healthy controls, indicating functioning (de-)recruitment of CBV associated with changes of feed vessel blood flow in healthy subjects (*R*^2^ = 0.12; *p* = 0.23) (Fig. 2C). CR was 29% in long COVID, 17% in COVID-19 patients and 70% in healthy volunteers (Fig. 2D). This finding suggests that the number of perfused capillaries in long COVID remains *fixed* and does not adequately respond to local variations of tissue metabolic demand. Associating the different symptoms with the capillary density in an explorative manner, revealed a statistically significant capillary rarefication in long COVID individuals presenting with neurocognitive dysfunction (38.50 [25.93–44.41] vs. 49.77 [43.40–64.30], *p* = 0.049; Supp. Table 1).

**Fig. 3:**
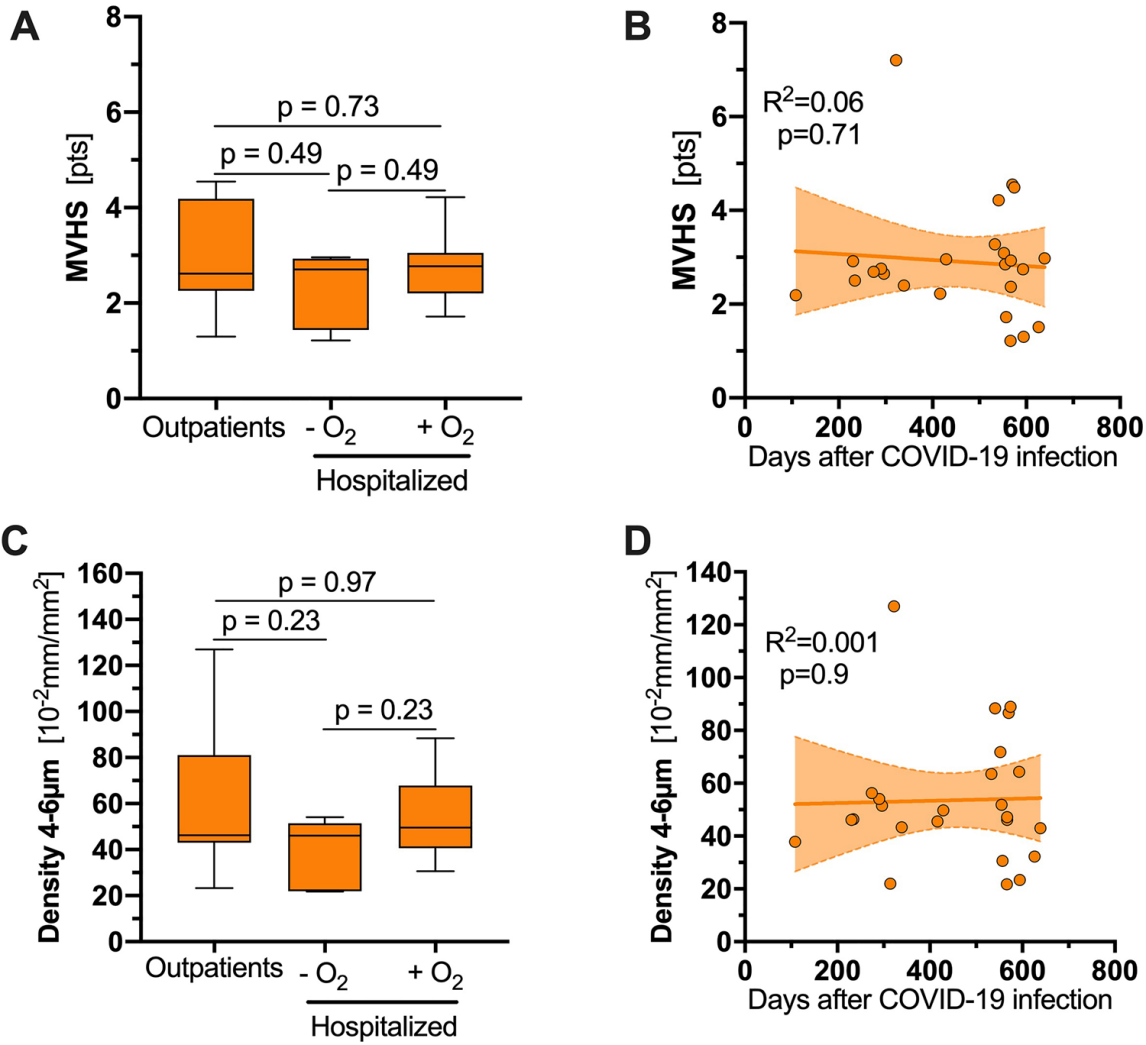
Sensitivity analyses in individuals with long COVID. **A**, **C** Boxplot of (**A**) MVHS and (**C**) density of long COVID individuals based on the clinical course of their acute COVID-19 infection. **B**, **D** Scatter dot plots and simple linear regression (slope) with 95% confidence intervals of (**B**) MVHS and (**D**) capillary density plotted against the days after the COVID-19 infection. *COVID*-19 Coronavirus disease 2019, *MVHS* microvascular health score, *ns* not significant

By further analyses, long COVID individuals showed no significant difference in MVHS or capillary density irrespective of outpatient or inpatient care—regardless of need for oxygen supplementation, during acute COVID-19 infection (Fig. 3A, C; Table 2). To assess recovery of microcirculatory parameters over time, we plotted MVHS and capillary density against the length of time between initial infection and presentation to the long COVID study outpatient clinic. Interestingly, no improvement of these parameters was documented, assuming capillary loss due to COVID-19 might be irreversible (Fig. 3B, D).

**Table 2:** Long COVID cohort divided with regard to the acute infection

Variable	Outpatient	Hospitalized without oxygen supplementation	Hospitalized with oxygen supplementation	*p* value*
Number of participants (*n*; %)	12	7	8	–
Female sex (*n*; %)	8 (66.7)	2 (28.6)	2 (25)	0.11
Age [years, median (IQR)]	54 (36–58)	44 (32–63)	59 (47–61)	0.35
BMI [kg/m^2^, median (IQR)]	26.3 (21.2–29.1)	26.6 (24–28)	28.04 (24.72–29.30)	0.67
Critical illness (*n*; %)	–	–	3 (37.5)	–
Days after COVID-19 infection	550 (236–588)	314 (290–566)	553 (474–559)	0.50
CCI score [median (IQR)]	0 (0–1)	0 (0)	0 (0)	0.16
Long COVID symptoms (*n*; %)				
> 1 Symptoms	12 (100)	5 (71.4)	6 (75)	0.15
Fatigue/Weakness	10 (83.3)	7 (100)	6 (75)	0.39
Dyspnea	12 (100)	5 (71.4)	5 (62.5)	0.08
Chest discomfort	4 (33.3)	2 (28.6)	0 (0)	0.19
Neurocognitive dysfunction	1 (8.3)	2 (28.6)	1 (12.5)	0.48
Persistent cough	2 (16.7)	0 (0)	0 (0)	0.26
Headache	1 (8.3)	0 (0)	1 (12.5)	0.65
Sublingual videomicroscopy [median (IQR)]				
PBR (µm)	2.15 (1.97–2.29)	2.12 (2.03–2.31)	2.16 (2.0–2.22)	0.98
Capillary density (10^−2^ mm/mm^2^)	46.30 (43.03–81.04)	46.16 (21.96–51.48)	49.55 (40.64–67.92)	0.36
VRBC_4–7 µm_ (µm/sec)	119.84 (106.85–131.87)	105.40 (94.89–126.47)	121.99 (103.88–151.22)	0.43
VRBC_≥10 µm_ (µm/sec)	129.82 (120–130.72)	123.24 (113.01–133.71)	122.86 (119.09–131.96)	0.87
MVHS (points)	2.62 (2.26–4.19)	2.70 (1.44–2.93)	2.77 (2.21–3.05)	0.67

*p values were calculated with the use of Kruskal–Wallis test among the three groups

## Discussion

To the best of our knowledge, the present in vivo study is the first to address the microvasculature of long COVID individuals in a detailed, diameter class-wise manner. The number of small capillaries, but not of the supply vessels, were markedly reduced, very similar to the pattern found during severe COVID-19 infection [4].

In addition, capillary recruitment of COVID long-term patients remains significantly reduced. While healthy controls succeed in maintaining a constant capillary VRBC (almost horizontal slope), long COVID patients as well as acute COVID-19 patients fail to do so (steeper slopes). This finding reflects a persistent fixed number of perfused capillaries, which is insensitive to local tissue variations of metabolic demand [8].

To what extent this capillary rarefication is mechanical or/and functional remains unclear. Pretorius et al. revealed common clotting pathologies in plasma of acute and long COVID patients, further supporting the existence of persistent microthrombi [10]. Besides that, an insufficient recovery of the initial inflammation accompanied by persistent immunological abnormalities might also be responsible for the observed capillary impairment [6]. Previous studies focusing on the pulmonary [11], exertional [12] and myocardial [13] microcirculation of long COVID individuals showed local impairment of the microcirculation. Our data complement these findings and point to a systemic and long-lasting capillary rarefication. This finding could possibly explain the functional impairment observed in long COVID syndrome. A subtle but exciting feature of the long-haul COVID group is, that the RBC velocities are the highest in our long COVID cohort (indicated by parallel shift of the slope to the upper right). We speculate that this finding could represent a compensatory mechanism to meet metabolic demands. Considering that the measurements were taken at physical rest, it is quite conceivable that this presumed compensatory mechanism is exhausted more quickly during exertion than in healthy individuals, possibly explaining long COVID symptoms. Here, we previously were able to present data on persistent exertional impairment caused by (micro-) circulatory reduction of the oxygen pulse during sequential, bicycle cardiopulmonary exercise test [12].

In the TUN-EndCOV Study, Charfeddine et al. reported a significant microvascular and endothelial dysfunction in a post-COVID cohort, evaluated by finger thermal monitoring after occlusion and reperfusion of the hand [14]. Specifically, long COVID individuals showed a higher endothelial quality index and a slower response to the reperfusion phase. This finding was partially reversed after oral intake of sulodexide, a highly purified mixture of glycosaminoglycans, that includes fast-moving heparin and dermatan sulfate [15]. Indeed, our data imply that long COVID individuals respond inadequately to local variations of tissue metabolic demand. Therefore, it is intriguing to speculate, that this delayed response after reperfusion might be the functional result of the observed capillary rarefication and the impaired capillary recruitment of the remaining capillaries in the long COVID individuals.

Regarding endothelial glycocalyx, previous studies have shown a trend towards partial recovery of the glycocalyx in the first months following the acute infection [16, 17]. Ikonomidis et al. evaluated PBR values of post-COVID individuals 12 months after infection, showing persistent glycocalyx impairment [18]. Here we demonstrate completely restored glycocalyx dimensions in long COVID individuals about 1.5 years after the acute infection. Mechanistically, glycocalyx thinning is tightly controlled, among others by the endothelium specific Angiopoietin(Angpt)/Tie2 system. We have shown that Angpt-2 activates heparanase release from the endothelium which leads to enzymatic degradation of the endothelial glycocalyx [19]. Indeed, we and others showed that both heparanase and Angpt-2 levels are elevated during the acute infection [4, 20]. Recent studies in long COVID reported normalized heparanase and Angpt-2 levels [21], consistent with restored glycocalyx dimensions. In this regard, our data once again highlight differential regulation of microcirculation parameters and glycocalyx dimensions, a finding we first observed in critically ill patients with bacterial sepsis [22].

We acknowledge some limitations. First, our pilot study had a small sample size and is mostly hypothesis-generating. Our study is observational and does not demonstrate causality between microvascular damage and post-COVID symptoms. Second, we included a historical cohort to better understand the microvascular changes. However, all microvascular parameters were assessed under the same conditions using the same GlycoCheck camera by the same experienced investigator. Third, plasma samples are not available in our long COVID patients, so we cannot perform further analyses of endothelial markers. Fourth, the capillary recruitment was calculated post-hoc in a per group analysis; meanwhile a per individual analysis has become available which might provide even more accurate results.

## Conclusion and outlook

Microvascular impairment appears to play a crucial role in the pathophysiology of both COVID-19 and post-COVID sequelae [2, 23]. In summary, our current data strongly suggest that COVID-19 leaves a persistent capillary rarefication even 18 months after infection. Whether, to what extent, and when the observed damage might be reversible remains unclear. If capillary rarefaction were to persist, additional cardiovascular disease (e.g., diabetes, hypertension) will be less well compensated and become symptomatic much earlier. It therefore seems important to understand the mechanisms of capillary rarefication in long COVID in detail. Here, we see a useful application for our µm-wise quantitative video microscopy [8] in characterizing study patients (enrichment) and monitoring response to potential therapies.

## Supplementary Information

Below is the link to the electronic supplementary material.

Supplementary file1 (DOCX 20 kb)

## Data Availability

The datasets used and/or analyses during the current study are available from the corresponding author on reasonable request.

## Abbreviations

Angpt-2: Angiopoietin-2
BMI: Body mass index
CCI: Charlson Comorbidity Index
COVID-19: Coronavirus disease 2019
CR: Capillary recruitment
IQR: Interquartile range
MVHS: Microvascular health score
RBC: Red blood cell
SOFA: Sequential Organ Failure Assessment
VRBC: Red blood cell velocity
