# Supplementary material for: Persistent capillary rarefication in long COVID syndrome
Source: Angiogenesis. 2022 Aug 11;26(1):53–61. doi: 10.1007/s10456-022-09850-9 (PMC9366128; doi:10.1007/s10456-022-09850-9)
Supplement: Supplementary file 1 — Supplementary file1 (DOCX 20 kb) [file 10456_2022_9850_MOESM1_ESM.docx]

**Persistent capillary rarefication in long COVID syndrome**

Irina Osiaevi^1^, Arik Schulze^1^, Georg Evers^1^, Kimon Harmening^1^, Hans Vink^2^, Philipp Kümpers^3^, Michael Mohr^1^*, Alexandros Rovas^3^*

^1^Department of Medicine A, Hematology, Oncology and Pulmonary Medicine, University Hospital Münster, 48149 Münster, Germany

^2^Department of Physiology, Cardiovascular Research Institute Maastricht, Maastricht University, Maastricht, The Netherlands

^3^Department of Medicine D, Division of General Internal and Emergency Medicine, Nephrology, and Rheumatology, University Hospital Münster, Albert-Schweitzer-Campus 1, 48149 Münster, Germany

*These authors contributed equally to this work and are both considered senior authors

**Supp. Table 1:** Capillary density (median (IQR) - measured in 10^-2^mm/mm^2^) of the long COVID cohort based on the presence (+) or absence (-) of different symptoms.

| **Long COVID symptoms** | **(+)** | **(-)** | ***p value*** |
| --- | --- | --- | --- |
| >1 symptoms | 46.43 (42.94-63.49) | 43.17 (31.06-67.38) | 0.77 |
| Fatigue/Weakness | 47.21 (39.15-63.49) | 44.23 (33.73-65.23) | 0.53 |
| Dyspnea | 46.82 (43.21-63.69) | 39.15 (31.45-62.95) | 0.49 |
| Chest discomfort | 57.04 (45.36-96.72) | 46.17 (35.04-55.18) | 0.16 |
| Neurocognitive dysfunction | 38.50 (25.93-44.41) | 49.77 (43.40-64.30) | **0.049** |
| Persistent cough | 86.27 | 46-43 (38.50-59.88) | 0.41 |
| Headache | 65.66 | 46.43 (38.50-59.88) | 0.69 |
